# Feasibility of edoxaban for asymptomatic cancer-associated thrombosis in Japanese patients with gastrointestinal cancer: ExCAVE study

**DOI:** 10.1186/s12885-022-10403-y

**Published:** 2022-12-16

**Authors:** Michio Nakamura, Atsushi Ishiguro, Masayoshi Dazai, Yasuyuki Kawamoto, Satoshi Yuki, Susumu Sogabe, Ayumu Hosokawa, Kentaro Sawada, Osamu Muto, Naoki Izawa, Koji Nakashima, Yoshiki Horie, Masataka Yagisawa, Shinya Kajiura, Takayuki Ando, Yosuke Mitsuhashi, Yu Sunakawa, Yasuka Kikuchi, Yoshito Komatsu

**Affiliations:** 1 Department of Gastroenterology, Sapporo City General Hospital, 1-1, Kita 11-jo Nishi 13-chome, Chuo-ku, Sapporo, 060-8648 Japan; 2 Department of Medical Oncology, Teine Keijinkai Hospital, Sapporo, Japan; 3 Department of Gastroenterology, Sapporo Medical Center NTT EC, Sapporo, Japan; 4 Division of Cancer Center, Hokkaido University Hospital, Sapporo, Japan; 5 Department of Gastroenterology and Hepatology, Hokkaido University Hospital, Sapporo, Japan; 6 Department of Medical Oncology, KKR Sapporo Medical Center, Sapporo, Japan; 7 Department of Clinical Oncology, University of Miyazaki Hospital, Miyazaki, Japan; 8 Department of Medical Oncology, Kushiro Rosai Hospital, Kushiro, Japan; 9 Department of Medical Oncology, Japanese Red Cross Akita Hospital, Akita, Japan; 10 Department of Clinical Oncology, St. Marianna University School of Medicine, Kawasaki, Japan; 11 Department of Gastroenterology, Japanese Red Cross Kitami Hospital, Kitami, Japan; 12 Department of Medical Oncology, Toyama University Hospital, Toyama, Japan; 13 Third Department of Internal Medicine, University of Toyama, Toyama, Japan; 14 Department of Surgery, IMS Sapporo Digestive Disease Center General Hospital, Sapporo, Japan; 15 Department of Diagnostic Imaging, Faculty of Medicine, Hokkaido University, Sapporo, Japan; 16 Department of Radiology, Sapporo Medical Center NTT EC, Sapporo, Japan

**Keywords:** Thrombosis, Edoxaban, Gastrointestinal cancer, Bleeding, Heparin

## Abstract

**Background:**

Although initial therapy with a parenteral anticoagulant is required before edoxaban, this strategy is frequently avoided in actual clinical practice because of its complexity. This study assessed the feasibility of edoxaban without initial heparin usage for asymptomatic cancer-associated thrombosis (CAT) in Japanese patients with gastrointestinal cancer (GIC) at high risk of bleeding.

**Methods:**

In this multicenter prospective feasibility study conducted at 10 Japanese institutions, patients with active GIC who developed accidental asymptomatic CAT during chemotherapy were recruited. Edoxaban was orally administered once daily without initial parenteral anticoagulant therapy within 3 days after detecting asymptomatic CAT. The primary outcome was the incidence of major bleeding (MB) or clinically relevant non-major bleeding (CRNMB) during the first 3 months of edoxaban administration.

**Results:**

Of the 54 patients enrolled from October 2017 to September 2020, one was excluded because of a misdiagnosis of CAT. In the remaining 53 patients, the primary outcome occurred in six patients (11.3%). MB occurred in four patients (7.5%), including gastrointestinal bleeding in three patients and intracranial hemorrhage in one patient. CRNMB occurred in two patients (3.8%), including bleeding from the stoma site and genital bleeding in one patient each. There were no deaths attributable to bleeding, and all patients who experienced MB or CRNMB recovered.

**Conclusions:**

The risk of bleeding after edoxaban without heparin pretreatment was acceptable, demonstrating new treatment options for asymptomatic CAT in patients with GIC.

**Supplementary Information:**

The online version contains supplementary material available at 10.1186/s12885-022-10403-y.

## Introduction

Cancer-associated thromboembolism (CAT) is a critical life-threatening complication among patients with cancer, occurring in approximately 7–15% of patients with cancer [1–4]. The Cancer-VTE Registry, a large-scale prospective cohort study in Japan, reported that VTE prevalence increased with progression in disease stage and that the VTE prevalence rates were 13.2 and 11.2% in patients with stage IV gastric and colorectal cancer, respectively [5]. Although several guidelines recommended low-molecular-weight heparin (LMWH) or direct oral anticoagulants (DOACs) for CAT [6–9], LWMH is not approved by Japanese public medical insurance for use as an anticoagulant against CAT. Therefore, DOACs are currently the most recommended treatments for CAT in Japan. Whereas DOACs have the advantages of oral administration and easy dosing, which might enhance patient compliance, they have been reported to carry a risk of gastrointestinal (GI) bleeding [10–12]. In particular, DOACs are not recommended as first-line treatments for patients with luminal GI cancer (GIC) with a primary tumor or active GI mucosal abnormalities (e.g., gastric/duodenal ulcer, gastritis, colitis, esophagitis) [8, 13]. Most recently, the National Comprehensive Cancer Network guidelines indicated that apixaban, edoxaban, or rivaroxaban are preferred for patients other than those with gastric or gastroesophageal lesions with higher bleeding rates [14].

When using DOACs in patients with GI malignancy, evaluating the risk–benefit balance in individual cases is essential. Of the three DOACs with direct factor Xa inhibitory effects, both apixaban and rivaroxaban are approved for the initial treatment period. However, edoxaban requires initial treatment with a parenteral anticoagulant such as unfractionated heparin (UFH), and there are no data regarding the safety and efficacy of edoxaban treatment without heparin pretreatment. In the ETNA-VTE Japan study, which provided real-world data about edoxaban use in Japan, it was reported that only 56.1% of patients received prior anticoagulant therapy before using edoxaban, and edoxaban alone was used in approximately half of the patients [15]. Edoxaban has a shorter time to active onset than apixaban and rivaroxaban [8], and heparin administration before edoxaban treatment may be omitted in patients with asymptomatic thrombosis who do not require urgent treatment. The use of UFH as an initial treatment requires inpatient management with continuous intravascular infusion, repeat venipuncture for monitoring (anti-factor Xa levels or activated partial thromboplastin time), and dose adjustment. In addition, inpatient treatment for patients with asymptomatic CAT is inconvenient and costly; thus, a clinical need to omit prior heparin administration before using edoxaban exists for patients with asymptomatic CAT.

In the ExCAVE study, a multicenter prospective feasibility study of edoxaban for asymptomatic CAT in Japanese patients with GIC and a high bleeding risk, we assessed whether initial treatment with UFH could be omitted in this setting.

## Patients and methods

### Study design

ExCAVE was a single-arm, multicenter prospective feasibility study. The enrollment period was from October 2017 to September 2020. The protocol was performed in accordance with the Declaration of Helsinki, Japanese ethical guidelines on clinical research, and Ethical Guidelines for Clinical Studies. The study was registered with the Japan Registry of Clinical Trials (jRCTs011180030) and the University Hospital Medical Information Network Clinical Trials Registry (protocol ID UMIN000028517) on June 23, 2017. Written informed consent was obtained from all patients. The institutional review board at each participating center approved the protocol.

### Patients

Patients with histologically confirmed GI malignancy who were undergoing systemic chemotherapy and who were newly diagnosed with incidental, asymptomatic deep-vein thrombosis (DVT) in the lower (popliteal, femoral, or iliac vein) or upper limbs (subclavian or internal jugular vein) or asymptomatic pulmonary embolism (PE) during chemotherapy were eligible to participate in this study. Incidental DVT or PE was detected on imaging performed for reasons other than clinical suspicion of venous thromboembolism (VTE). Patients with symptomatic CAT were excluded from the study. In the case of DVT, signs such as erythema, warmth, pain, swelling, tenderness, and pain during dorsiflexion of the legs were defined as “symptomatic,” and in the case of PE, signs such as sudden dyspnea, frequent breathing, tachypnea, fainting, hypotension, and hypoxia were defined as “symptomatic.” New DVT diagnosis was defined as newly occurring thrombosis in patients with a confirmed serum D-dimer level of ≤1.2 μg/mL before study enrollment. Additionally, patients with serum D-dimer levels above 1.2 μg/mL who were confirmed not to have thromboses using chest and abdominal contrast-enhanced computed tomography (CT) or venous ultrasonography before enrollment were also considered as new-onset DVT and were permitted for enrollment in the present study. The other inclusion criteria and exclusion criteria–including patients’ clinical characteristics, issues related to anticoagulant treatment, bleeding risk, and problems standard from clinical trials of anticoagulant agents–are listed in the Supplementary Appendix.

### Trial treatment

Edoxaban was administered without prior heparin administration in this study, orally at a fixed dose once daily and at a lower dose (30 mg once daily) in patients with creatinine clearance (CrCl) of ≤50 mL/min or body weight of ≤60 kg, as well as in patients receiving concomitant treatment with potent P-glycoprotein inhibitors. In all patients, treatment with edoxaban continued for at least 3 months, and the treating physician determined the duration beyond 3 months.

### Outcome assessment

The primary outcome was a composite of major bleeding (MB) and clinically relevant non-major bleeding (CRNMB) during the 3-month study period. MB was defined as clinically overt bleeding associated with a decrease in the hemoglobin level of at least 2 g/dL, the transfusion of at least two units of red cells, bleeding occurring at a critical site (e.g., intracranial, intraspinal, intraocular, retroperitoneal, intraarticular, pericardial, or intramuscular with compartment syndrome), or fatal bleeding as per the criteria of the International Society on Thrombosis and Haemostasis [16]. CRNMB was defined as clinically overt bleeding not meeting the criteria for MB but requiring medical attention, causing discomfort, or impairing activities of daily living [17]. The criteria for evaluating VTE and the secondary endpoints are provided in the Supplementary Appendix. Regarding the imaging studies utilized for the evaluation of thrombosis, the studies conducted immediately before the start of edoxaban administration were used as baseline and those conducted within 28 days before edoxaban administration were allowed. In addition, the imaging studies used for the 3-month evaluation included those conducted up to 2 weeks before and after the 3-month follow-up. The results of contrast-enhanced CT studies performed to determine the efficacy of treatment during the 3-month study period were also included for the evaluation of thrombosis. Regarding the validity of the CAT diagnosis and determination of thrombus recurrence, a single independent radiological specialist, without additional clinical information, reviewed the imaging data that triggered the diagnosis of CAT and the subsequent imaging data. Furthermore, the rate of thrombus reduction was defined as the proportion of patients in whom the thrombus disappeared or exhibited a reduction in volume. The rate of thrombus control was defined as the proportion of patients in whom the thrombus disappeared or did not increase in volume.

### Safety and tolerability

Study physicians assessed safety and tolerability at each visit for chemotherapy. Whether adverse effects were related to the study drug was evaluated by a study physician when reporting each event.

### Statistical analysis

For categorical variables, the proportion was calculated, and contingency tables were created. The chi-squared test was used to compare categorical variables, and Fisher’s exact test was used when the frequency of any cell of the contingency table was ≤5. For continuous variables, summary statistics (mean, standard deviation) were calculated. For each risk factor for bleeding and VTE recurrence, the incidence (95% confidence interval [CI]) was estimated. In this study, if the one-side range width of the 95% CI of the primary outcome was within 9%, it was judged that the accuracy was sufficient for estimating the primary outcome. In the present study, we assumed that the incidence of MB and CRNMB would be 12%, based on the subset analysis of the Hokusai VTE Cancer study [18]. Calculation using the Wilson score interval method indicated an estimated sample size of 80 to maintain a one-sided 95% CI of 8.9%, a two-sided alpha level of 0.05, power of 80%, and an effect size of 0.317. We estimated that 100 cases would be needed to achieve the required number of subjects during the patient accrual (2 years) and follow-up periods (3 months) assuming 20% attrition. The planned study enrollment period was 2 years, which was considered feasible to recruit 100 patients across 10 Japanese participating facilities based on pre-surveillance. In addition, the study observation period was set to 3 months based on the aim to evaluate the short-term impact of edoxaban without initial treatment, and not its long-term effects. Time-to-event curves were calculated using the Kaplan–Meier method. Statistical significance was set at *P* < 0.05. Statistical calculations were performed using SPSS for Macintosh (release 24.0; SPSS Inc., Chicago, IL, USA) and R for macOS (version 4.1.0, The R Foundation for Statistical Computing Platform).

## Results

### Patient characteristics

**Fig. 1:**
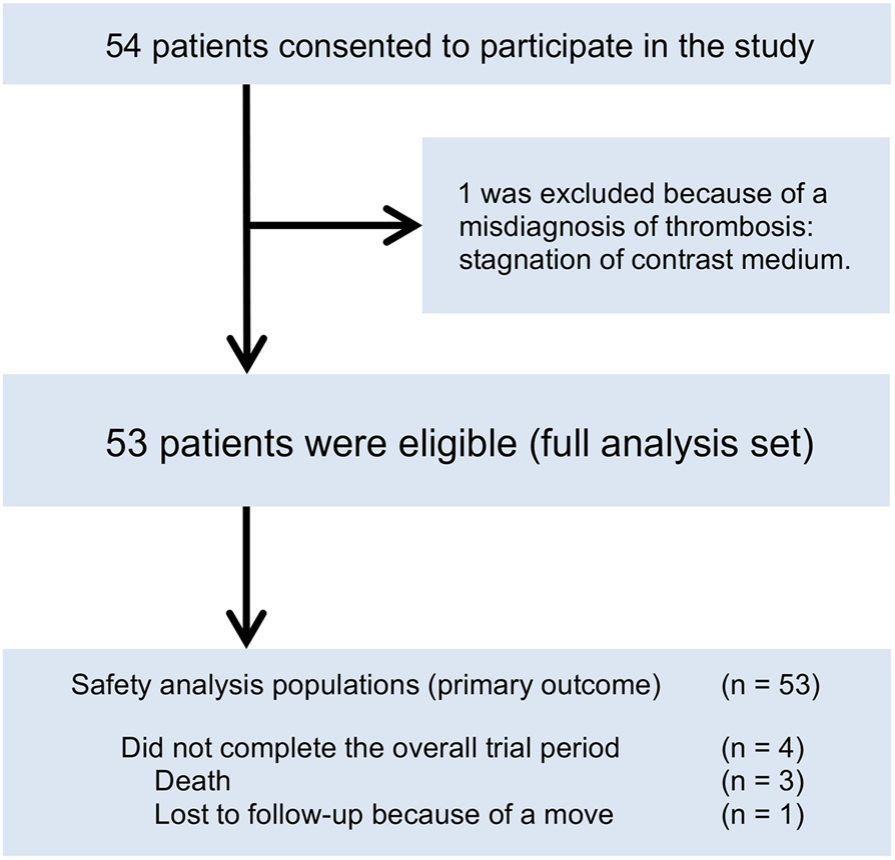
Flow chart of patient enrollment. Adults presenting with new asymptomatic venous thromboembolism (VTE), deep vein thrombosis (DVT), or pulmonary thromboembolism (PE) during systemic chemotherapy after the histological diagnosis of gastrointestinal cancer were eligible to participate in the study. The primary study endpoint (safety) was evaluated in patients who received the study drug at least once

**Fig. 2:**
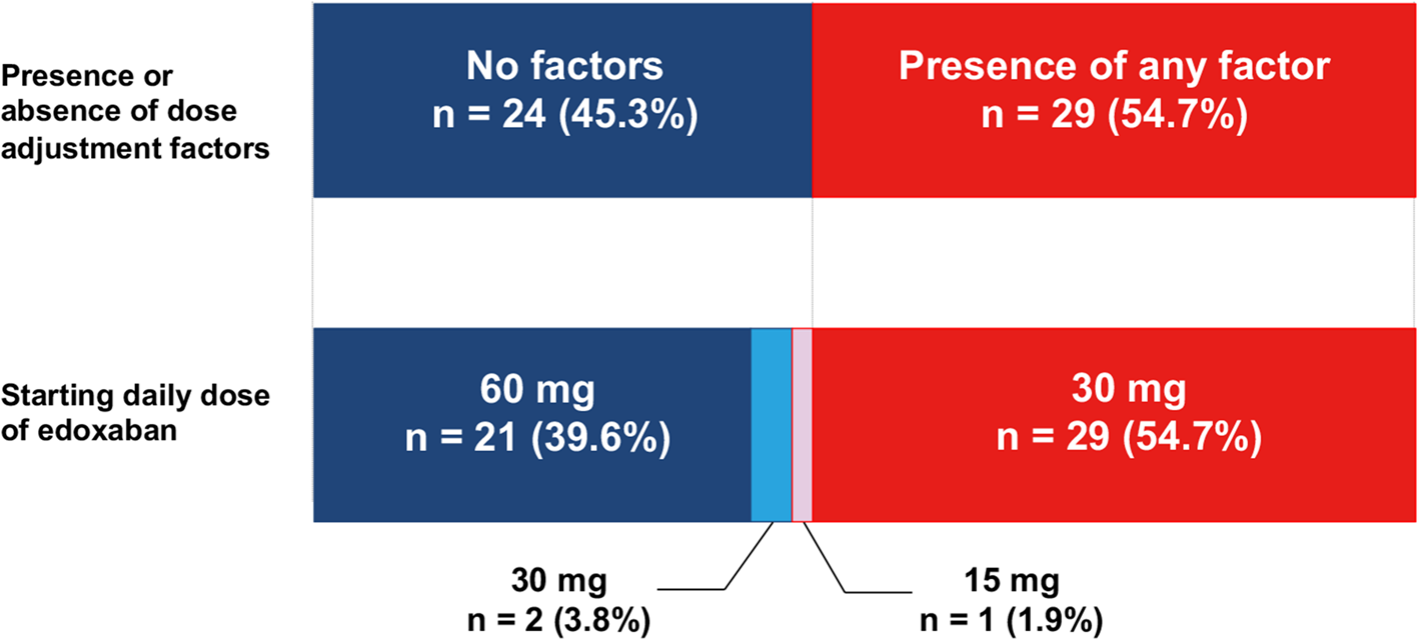
Edoxaban medication status. Starting edoxaban daily dose according the presence of the following dosage adjustment factors: body weight ≤ 60 kg, creatinine clearance ≤50 mL/min, and concomitant use of P-glycoprotein inhibitors (e.g., quinidine)

From October 2017 to September 2020, 54 patients were enrolled at 10 Japanese institutions (Fig. 1). One patient was excluded because of a misdiagnosis of CAT. The full analysis set (FAS) for assessing the primary outcome included 53 patients (28 men, 25 women; age, 38–83 years; median age, 65.3 years). Table 1 presents the baseline characteristics of the FAS. Regarding the daily dose at the start of edoxaban treatment, 1.9, 58.5, and 39.6% of patients received doses of 15, 30, and 60 mg, respectively. Details about the dosage levels and dose adjustment factors (DAFs) are presented in Fig. 2. All 29 patients with DAFs received the reduced edoxaban dose of 30 mg. Of the patients without DAFs (*n =* 24, 45.3%), 87.5% (*n =* 21) received edoxaban 60 mg. Thus, 50 patients (94.3%) received edoxaban at the recommended dosage. Of the three patients (5.7%) who received a non-recommended dosage: all three received a reduced dose (30 mg for two patients [3.8%] and 15 mg for one patient [1.9%]).

**Table 1:** Baseline patient characteristics

Characteristics (*n =* 53)	Result
Male sex, n (%)	28 (53)
Age	
Median, years (range)	65.3 (38–83)
≥ 75 years, n (%)	9 (17.0)
ECOG PS, n (%)	
0	38 (71.7)
1	14 (26.4)
2	1 (1.9)
Body weight	
Mean ± SD, kg	59.7 ± 8.9
≤ 60 kg, n (%)	28 (52.8)
Platelet count of 50,000–100,000/μL, n (%)	2 (3.8)
Pretreatment creatinine clearance, n (%)	
≤ 50 mL/min	2 (3.8)
> 50–80 mL/min	29 (54.7)
> 80 mL/min	22 (41.5)
VTE diagnosis, n (%)	
PE with or without DVT	11 (20.8)
Proximal DVT	40 (75.5)
Distal DVT	4 (7.5)
Location of DVT, n (%)	
Internal jugular vein	7 (13.2)
Subclavian vein	12 (22.6)
Axillary vein	1 (1.9)
Superior vena cava	13 (24.5)
Portal vein	1 (1.9)
Superior mesenteric vein	1 (1.9)
Ovarian vein	4 (7.5)
Femoral vein	1 (1.9)
Soleal vein	3 (5.7)
Anterior tibial vein	1 (1.9)
Posterior tibial vein	1 (1.9)
Peroneal vein	2 (3.8)
Currently receiving cancer treatment, n (%)	
Chemotherapy for advanced/metastatic cancer	48 (90.6)
Neo-adjuvant chemotherapy	2 (3.8)
Adjuvant chemotherapy	3 (5.7)
Primary tumor type, n (%)	
Colorectal	30 (56.6)
Esophageal	6 (11.3)
Gastric	6 (11.3)
Pancreatic	9 (17.0)
Other	2 (3.8)
CVC placement, n (%)	
No	6 (11.3)
Yes	47 (88.7)

Abbreviations: *CVC* Central venous catheter, *DVT* Deep-vein thrombosis, *ECOG PS* Eastern Cooperative Oncology Group performance status, *PE* Pulmonary embolism, *VTE* Venous thromboembolism

### Primary outcome

**Fig. 3:**
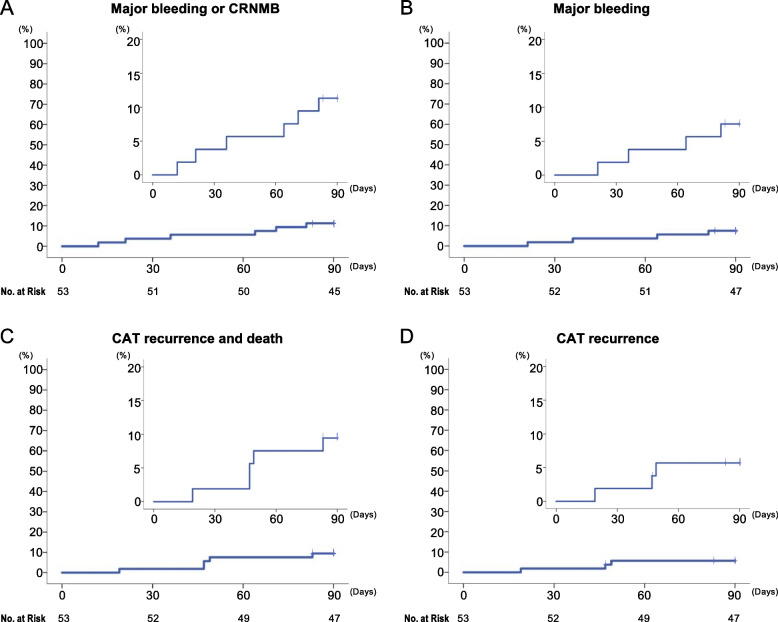
Primary and secondary study outcomes. Kaplan–Meier curves of the primary and secondary outcomes within 3 months. (**A)** MB or CRNMB, (**B**) MB alone, (**C**) CAT recurrence and death, and (**D**) CAT recurrence alone. The inset presents the same data on an enlarged y-axis. CAT, cancer-associated thrombosis; CRNMB, clinically relevant non-major bleeding; MB, major bleeding

The time to occurrence of the primary outcome is presented in Fig. 3, and the characteristics of these events are presented in Table 2. The primary endpoint occurred in six patients (11.3%; Fig. 3A). Specifically, MB occurred in four patients (7.5%; Fig. 3B), including three cases of gastrointestinal bleeding and one case of intracranial hemorrhage. CRNMB occurred in two patients (3.8%), including one patient each with bleeding from the stoma and genital bleeding. There were no deaths attributable to bleeding, and all patients recovered. The actual number of registered cases in this study was 53, which did not reach the expected number of 100. However, the one-side range of the 95% CI based on the binomial distribution of the primary event occurrence rate of 11.3% in this study was 9.38%, which was similar to the initially assumed rate, and it was appropriate for accurately evaluating the primary outcome.

**Table 2:** Primary outcome

Outcome (*n =* 53)	No. of patients (%)
MB or CRNMB	6 (11.3)
MB	4 (7.5)
Criteria defining MB^a^	
Clinically overt and decrease in the hemoglobin level of ≥2 g/dL over 24 h	2 (3.8)
Clinically overt and transfusion of ≥2 units of packed red cells	2 (3.8)
Clinically overt and located at a critical site (e.g., intracranial, retroperitoneal)	2 (3.8)
Clinically overt and contributing to death	0 (0)
Sites of MB, no./total no. (%)	
GI	3/4 (75)
Upper GI	1/4 (25)
Lower GI	1/4 (25)
Site unknown	1/4 (25)
Intracranial	1/4 (25)
Clinical presentation severity category^b^, no./total no. (%)	
Category 1	1/4 (25)
Category 2	1/4 (25)
Category 3	2/4 (50)
Category 4	0
CRNMB	2 (3.8)
Criteria defining CRNMB	
Overt bleeding requiring medical intervention	0
Unscheduled contact with a physician	0
Interruption of edoxaban	2 (3.8)
Discomfort or impairment of activities of daily living	0
Site of CRNMB, no./total no. (%)	
Gastrointestinal (stoma)	1 (50)
Genitourinary (vagina)	1 (50)

Abbreviations: *CRNMB* Clinically relevant non-major bleeding, *GI* Gastrointestinal, *MB* Major bleeding

^a^One patient had three reasons: decreased hemoglobin levels, transfusion of red cells, and critical site bleeding

^b^Classification of the clinical presentation of major bleeding events: Category 1, bleeding events presenting without any clinical emergency; Category 2, all bleeding events requiring certain measures without necessitating urgent measures that could not be classified to any of the other three categories; Category 3, bleeding events causing a major medical emergency; such as hemodynamic instability, or cerebral bleeding presenting with neurologic symptoms; and Category 4, bleeding events that were fatal before or almost immediately after entering the hospital

### Secondary outcomes

The combined endpoint of thrombus recurrence and death occurred in five patients (9.4%; Fig. 3C). Recurrent CAT occurred in three patients (5.7%; Fig. 3D), including one patient with newly symptomatic PE and two patients with apparent increases in the thrombus volume versus baseline. Meanwhile, three patients died (5.7%), including two deaths attributable to cancer progression and one sudden death of unknown cause. Seven patients had multiple thrombi, and the worst thrombus evaluation results were included in the study analyses for these cases. According to the investigator’s assessment and the central review, the rates of thrombus reduction were 83.0% (44/53) and 75.5% (40/53), and the rates of thrombus control were 92.5% (49/53) and 83.0% (44/53). Among the patients in whom the thrombi disappeared, the median time to thrombus disappearance was 63.0 ± 17.9 (range, 25–98) days according to the investigator’s assessment and 63.5 ± 19.4 (range, 25–104) days according to the central review, respectively (Table 3). The rate of concordance in thrombosis assessment between the investigator’s assessment and the central review was 64.1% (34/53).

**Table 3:** Changes of the thrombus volume during 3 months of edoxaban administration

Thrombus^*^	No. of patients (%)
**Investigators’ assessment (*n =*** **53)**	
Disappeared	35 (66.0)
Improved	9 (17.0)
No change	5 (9.4)
Exacerbation	3 (5.7)
Not evaluable	1 (1.9)
TRR^a^	44 (83.0)
TCR^b^	49 (92.5)
Time to blood clot disappearance (days, *n =* 35), median ± SD (range)	63.0 ± 17.9 (25–98)
**Central review (*n =*** **53)**	
Disappeared	26 (49.1)
Improved	14 (26.4)
No change	4 (7.5)
Exacerbation	4 (7.5)
Not evaluable	5 (9.4)
TRR^a^	40 (75.5)
TCR^b^	44 (83.0)
Time to blood clot disappearance (days, *n =* 26), median ± SD (range)	63.5 ± 19.4 (25–104)

^*^Seven patients had multiple thrombi, and the worst thrombus evaluation results were included in analyses for these cases. Both the investigator’s assessment and central review adjudicated the evaluation of changes in the thrombus volume into the following categories:

*Exacerbation*, new thrombus formation or apparent increase in the thrombus volume versus baseline. An increase in the thrombus diameter of 4 mm or more was defined as an increase.; *No change*, neither exacerbation nor improvement.; *Improved*, loss of blood clots, or apparent reduction of the thrombus volume versus baseline. Shrinkage was indicated by a reduction of the thrombus volume of at least 50%.; *Disappeared*, the absence of a thrombus present at baseline. ^a^ TRR, thrombus reduction rate; ^b^ TCR, thrombus control rate

### Continuation or discontinuation of treatment and treatment duration

Of the 53 patients included in the FAS, 10 (18.9%) discontinued treatment during the study period. The reasons for discontinuation were bleeding-related AEs in four patients, thrombus disappearance in four patients, and primary cancer exacerbation in two patients. The mean duration of edoxaban treatment during the observation period was 81.1 ± 18.7 days, including durations of 84.6 ± 13.3 and 53.5 ± 31.2 days in patients without (*n =* 47) and with bleeding events (*n =* 6), respectively.

### Subgroup analyses

There was no statistically significant difference in outcomes according to the presence of DAF (Supplementary Fig. S1), edoxaban administration dose (15 mg vs. 30 mg vs. 60 mg; Supplementary Fig. S2), primary cancer site (Supplementary Fig. S3), renal function (CrCl: ≤50 mL/min vs. > 50–80 mL/min vs. > 80 mL/min; Supplementary Fig. S4), body weight (≤60 kg vs. > 60 kg; Supplementary Fig. S5), and age (< 75 years vs. ≥75 years old; Supplementary Fig. S6).

### Adverse events

The adverse events reported in this study are presented in Table 4. The most common bleeding event was epistaxis, occurring in seven patients (13.2%), although no events were serious.

**Table 4:** Adverse events during the entire observation period

Events	*n =* 53	
	Any grade^a^	Grade 3 or higher
	n (%)	n (%)
Bleeding events (overall)	15 (28.3)	3 (5.7)
Thalamic bleeding	1 (1.9)	1 (1.9)
Epistaxis	7 (13.2)	0
Gingival bleeding	1 (1.9)	0
Tracheal bleeding	1 (1.9)	0
Upper gastrointestinal bleeding	1 (1.9)	1 (1.9)
Lower gastrointestinal bleeding	2 (3.8)	0
Gastrointestinal bleeding (site unknown)	1 (1.9)	1 (1.9)
Genital bleeding	1 (1.9)	0
Anal bleeding	2 (3.8)	0
Hematuria	2 (3.8)	0
Laboratory test abnormalities (overall)	18 (34.0)	8 (15.1)
Anemia	7 (13.2)	2 (3.8)
Leukopenia	6 (11.3)	1 (1.9)
Neutropenia	10 (18.9)	7 (13.2)
Thrombocytopenia	1 (1.9)	0
Prolonged INR	4 (7.5)	0
Elevated AST	1 (1.9)	0
Elevated ALT	1 (1.9)	0
Hypoalbuminemia	1 (1.9)	0
Hypomagnesemia	1 (1.9)	0
Elevated serum creatinine	1 (1.9)	0
Proteinuria	2 (3.8)	0
Non-hematologic AE (overall)	23 (43.4)	3 (5.7)
Anorexia	5 (9.4)	1 (1.9)
Alopecia	1 (1.9)	0
Constipation	2 (3.8)	0
Cough	1 (1.9)	0
Diarrhea	5 (9.4)	1 (1.9)
Dysgeusia	6 (11.3)	0
Edema limbs	2 (3.8)	0
Fatigue	3 (5.7)	0
Hand-foot skin reaction	2 (3.8)	0
Headache	1 (1.9)	0
Hiccups	1 (1.9)	0
Hoarseness	2 (3.8)	0
Hyperpigmentation	1 (1.9)	0
Hypertension	2 (3.8)	2 (3.8)
Malaise	4 (7.5)	0
Nausea	3 (5.7)	0
Peripheral neuropathy	4 (7.5)	0
Pruritus	1 (1.9)	0
Stomatitis	2 (3.8)	0
Vomiting	1 (1.9)	0

^a^Grading was evaluated according to Common Terminology Criteria for Adverse Events v4.03

Abbreviations: *AE* Adverse event, *AST* Aspartate aminotransferase, *ALT* Alanine aminotransferase, *INR* Prothrombin time–international normalized ratio

## Discussion

In this challenging ExCAVE study, which evaluated the 3-month safety and efficacy of edoxaban without prior heparin administration in patients with GIC at high risk of bleeding, the incidence rates of MB or CRNMB, thrombus exacerbation or recurrence by either the central review or the investigator’s assessment, and all-cause death were 11% (*n =* 6), 9% (*n =* 5), and 6% (*n =* 3), respectively. On the other hand, in the analysis of events that occurred during the first 6 months in the Hokusai VTE Cancer study, which included patients who were given heparin administration before edoxaban initiation, the incidence rates of MB or CRNMB, VTE recurrence, and death were 16, 7, and 27%, respectively [17].

The Hokusai VTE Cancer study evaluated the safety and efficacy of edoxaban at 6 months; therefore, its results cannot be directly compared with the present study. However, a subset analysis on supplement data of the Hokusai VTE Cancer study found that the event-free survival rate at 3 months was 78% (407/522). In the present study, the rate of event-free survival, defined as the absence of bleeding, thrombus recurrence, and death, was 85% (45/53), suggesting that omitting heparin pretreatment may yield results comparable to those previously reported. The observation period in this study was 3 months, and the long-term safety of this strategy was not verified. However, no severe adverse events were observed during the observation period.

Edoxaban is the only fixed dose orally disintegrating DOAC tablet that can be taken once daily without scheduled titration. In addition, dose reduction criteria have been established for this drug to reduce the risk of bleeding, and the dose can be easily adjusted according to patients’ body weight and renal function. However, a single-drug approach using edoxaban without prior heparin administration is currently deprecated [6–9, 14]. In the Hokusai-VTE study, a 5-day course of initial treatment with heparin (enoxaparin or UFH), a global standard that was demonstrated as useful at the time, was incorporated into the study design. Since that study, prior administration of heparin has been recommended for treatment with edoxaban and the package insert for edoxaban states that edoxaban should be administered after appropriate initial treatment with a parenteral anticoagulant such as UFH. It is beneficial for patients to have edoxaban as an additional dose-adjustable DOAC option available without prior heparin administration for asymptomatic thrombosis.

Although several reports have discussed the efficacy and safety of DOACs for CAT [17–19], few studies focused on incidental asymptomatic CAT. For example, in the Sapporo CAT study, our previously reported observational study of CAT, only 43% of patients were treated for asymptomatic thrombosis [2]. Conversely, Gary et al. reported that asymptomatic venous thrombotic events were associated with poor survival [20]. However, inpatient treatment for asymptomatic thrombosis is excessive because heparin administration requires hospitalization, which imposes a heavy burden on patients in terms of cost. It has also been reported that rivaroxaban and edoxaban are less expensive than LMWH for the treatment of CAT, and DOACs are effective options as anticoagulant therapies for asymptomatic CAT even from the viewpoint of cost [21].

Generally, compared to the risk profile of dalteparin, edoxaban [11] and rivaroxaban [19] carry higher risks of MB in patients with GIC, and these DOACs should be used with caution in patients with unresected intraluminal tumors. By contrast, apixaban has been reported to have a bleeding rate equivalent to that of dalteparin in the treatment of CAT in patients with GIC [22]. Moreover, a report indicated that apixaban has the most favorable safety profile (MB risk) in a head-to-head comparison of three DOACs (dabigatran, apixaban, and rivaroxaban) [23]. However, no clinical trials have directly compared the safety profiles of DOACs in the treatment of CAT, and the safest DOAC for patients with CAT and GIC is unclear. Even when using apixaban, care should be taken when in patients with CAT and active GIC. That is, appropriate dose adjustment for at-risk patients is of paramount importance in bleeding risk management when using DOACs. In fact, more than half of the patients in this trial had at least one DAF, and all such patients underwent adequate dose adjustment to 30 mg.

Although no statistically significant difference was observed in this study, the frequency of bleeding events was slightly higher in the dose-adjusted group, suggesting that the frequency of bleeding might have increased further without dose reduction in patients with risk factors for bleeding, such as low body weight or low CrCl. In addition, in the present study, it is also important to note that PE occurred in one patient who did not undergo dose adjustment. In this patient, a dose of 60 mg should have been administered, but the dose was reduced to 30 mg at the attending physician’s discretion, indicating that the inappropriate dose reduction could be detrimental to the patients. The incidence of under-dosing of DOACs was investigated in the RIETE registry, which enrolled patients with VTE receiving DOACs [24]. In that study, 17% of patients receiving rivaroxaban and 50% of those receiving apixaban received a lower than recommended dose, far exceeding the rate in the present study (5.7%). Low inappropriate doses during initial therapy represent an essential advantage for edoxaban, for which the dose-setting criteria are clear.

The results of large-scale randomized controlled trials of apixaban [25] and rivaroxaban [26] as primary prophylactic treatments for CAT have been reported in recent years, but clinical studies on the prophylactic efficacy of edoxaban for CAT have not yet been conducted. The reason may be that prior heparin administration is recommended before edoxaban administration, and if heparin pretreatment is not required, edoxaban can also be considered as an option for prophylactic treatment. It has been reported that 75% of MB events associated with DOACs occur in patients with unresected GI tumors [27]. In other words, edoxaban can be used in cases in which the primary lesion was surgically removed. In addition, edoxaban, which is an orally disintegrating formulation, can be an easy-to-use drug for CAT prevention, especially during the postoperative period, even in cases of GIC in which the feeding condition is poor because of loss of appetite after surgery.

This study has notable limitations. First, this was a single-arm, prospective, interventional study without a control arm. Evaluating whether heparin administration could be omitted would require the inclusion of patients receiving prior heparin administration as a control arm. However, this approach was challenging in the present study because participants with asymptomatic CAT were less likely to accept heparin pretreatment, which required hospitalization. In Japan, LMWH, which is used as standard treatment for CAT in Europe and the United States, cannot be used due to insurance coverage. Therefore, it was not possible to compare LMWH and edoxaban in a randomized controlled trial setting in Japan. Second, the sample size was small, as the number of enrolled patients was approximately half of the target. One reason for the slow patient accrual was that this was a challenging clinical study targeting GIC, which carries a high risk of bleeding, and the COVID-19 pandemic may have also affected patient recruitment. However, as mentioned previously, the obtained results were similar to the accuracy assumed for evaluating the primary outcome, and it is considered that the results have specific implications. Third, the observation period was short (3 months), and the long-term effects have not been evaluated. Given that the purpose of the present study was to evaluate feasibility only at the early stage of the introduction of edoxaban without heparin, we used 3 months, not 6 months, as the time of evaluation. The protocol allowed each attending physician to decide whether to continue treatment or change the drug after 3 months. Further consideration of the long-term impact of this strategy is warranted. Fourth, the concordance rate was low between the thrombosis assessments performed by the investigators and the central review panel. Specifically, the difference between the investigator’s assessment and the central review tended to be more conspicuous for the determination of thrombus disappearance and improvement compared with the decision of exacerbation or no change in the thrombus. In most clinical studies using anticoagulants, the recurrence of thrombosis is often the endpoint and few studies have evaluated the state of thrombosis included in the present study. It should be noted that the discordance between the local and central evaluations may occur in studies where the disappearance of thrombus is defined as the endpoint to evaluate the efficacy of treatment for thrombosis.

## Conclusion

To our knowledge, this is the first study to evaluate the feasibility of edoxaban without initial heparin usage. Although the single-drug approach with edoxaban is an acceptable new treatment option for asymptomatic CAT, further studies are warranted to determine whether prior heparin treatment is essential in patients who are administered with edoxaban and to identify patients in whom prior heparin treatment can be omitted.

## Supplementary Information

**Additional file 1.**

**Additional file 2.**

**Additional file 3.**

**Additional file 4.**

**Additional file 5.**

**Additional file 6.**

**Additional file 7.**

## Data Availability

All data generated or analyzed during the current study are available from the corresponding author on reasonable request.

## Abbreviations

CAT: Cancer-associated thrombosis
CI: Confidence interval
CrCl: Creatinine clearance
CRNMN: Clinically relevant non-major bleeding
CT: Computed tomography
DAF: Dose adjustment factor
DOAC: Direct oral anticoagulant
DVT: Deep-vein thrombosis
FAS: Full analysis set
GI: Gastrointestinal
GIC: Gastrointestinal cancer
LMWH: Low-molecular-weight heparin
MB: Major bleeding
PE: Pulmonary embolism
UFH: Unfractionated heparin
VTE: Venous thromboembolism
