# Supplementary material for: Feasibility of edoxaban for asymptomatic cancer-associated thrombosis in Japanese patients with gastrointestinal cancer: ExCAVE study
Source: BMC Cancer. 2022 Dec 16;22:1322. doi: 10.1186/s12885-022-10403-y (PMC9757916; doi:10.1186/s12885-022-10403-y)
Supplement: Supplementary file 1 — Additional file 1. [file 12885_2022_10403_MOESM1_ESM.docx]

Supplemental Appendix

This appendix has been provided by the authors to give readers additional information about their work.

Supplement to: Nakamura M, Ishiguro A, Dazai M, et al. The feasibility of edoxaban for asymptomatic cancer-associated thrombosis in Japanese gastrointestinal cancer patients (ExCAVE study).

Supplemental Appendix Table of Contents

Contents

**Committees and investigators** 3

**Writing committee and affiliations** 3

**Executive committee** 3

**Coordinating management practitioner** 3

**Efficacy/safety evaluation committee** 3

**Data monitoring committee** 3

**Academic research organization** 3

**Investigators and study centers** 3

**Inclusion criteria** 5

**Exclusion criteria** 6

**Primary outcomes** 8

**Secondary outcomes** 8

**Index event and outcome event definitions** 9

**Bleeding events: MB** 9

**Categories of MB** 9

**Bleeding events: CRNMB** 9

**Nuisance (not clinically relevant) bleeding events** 9

**Evaluation of efficacy** 10

# **Committees and investigators**

## **Writing committee and affiliations**

The writing committee consisted of all authors listed for this article. The affiliations of the writing committee members are as follows:

Department of Gastroenterology, Sapporo City General Hospital, Sapporo, Japan (M.N.); Department of Medical Oncology, Teine Keijinkai Hospital, Sapporo, Japan (A.I.); Department of Gastroenterology, Sapporo Medical Center NTT EC, Sapporo, Japan (M.D.); Division of Cancer Center, Hokkaido University Hospital, Sapporo, Japan (Y.K.); Department of Gastroenterology and Hepatology, Hokkaido University Hospital, Sapporo, Japan (S.Y.); Department of Medical Oncology, KKR Sapporo Medical Center, Sapporo, Japan (S.S.); Department of Clinical Oncology, University of Miyazaki Hospital, Miyazaki, Japan (K.N., A.H.); Department of Medical Oncology, Kushiro Rosai Hospital, Kushiro, Japan (K.S.); Department of Medical Oncology, Japanese Red Cross Akita Hospital, Akita, Japan (O.M.); Department of Clinical Oncology, St. Marianna University School of Medicine, Kawasaki, Japan (Y.H., N.I., Y.S.); Department of Gastroenterology, Japanese Red Cross Kitami Hospital, Kitami, Japan (M.Y.); Department of Medical Oncology, Toyama University Hospital, Toyama, Japan (S.K.); Third Department of Internal Medicine, University of Toyama, Toyama, Japan (T.A.); Department of Surgery, IMS Sapporo Digestive Disease Center General Hospital, Sapporo, Japan (Y.M.); Department of Diagnostic Imaging, Faculty of Medicine, Hokkaido University, Sapporo, Japan (Y.K.); Department of Biostatistics, Yokohama City University School of Medicine (T.Y.)

## **Executive committee**

Yoshito Komatsu (chair)

Michio Nakamura (co-chair)

## **Coordinating management practitioner**

Michio Nakamura

## **Efficacy/safety evaluation committee**

Mototsugu Kato

Mitsutoshi Kurosawa

Takumi Oomura

## **Data monitoring committee**

Kayoko Iuchi

Mari Sekiguchi

## **Academic research organization**

HGCSG

## **Investigators and study centers**

Principal investigators

Teine Keijinkai Hospital (14 patients) – A. Ishiguro; Hokkaido University Hospital (11 patients) – Y. Komatsu, Y. Kawamoto, S. Yuki; Sapporo City General Hospital (11 patients) – M. Nakamura; Sapporo Medical Center NTT EC (6 patients) – M. Dazai; University of Miyazaki Hospital (3 patients) – A. Hosokawa; St. Marianna University School of Medicine (3 patients) – Y. Sunakawa; KKR Sapporo Medical Center (2 patients) – S. Sogabe; Toyama University Hospital (2 patients) – T. Ando, S. Kajiura; Japanese Red Cross Akita Hospital (1 patient) – O. Muto; IMS Sapporo Digestive Disease Center General Hospital (1 patient) – Y. Mitsuhashi

# **Inclusion criteria**

Adult subjects presenting with newly detected asymptomatic venous thromboembolism (VTE), deep vein thrombosis (DVT,) or pulmonary thromboembolism (PE) during systemic chemotherapy in patients who have been histologically diagnosed with gastrointestinal cancer were eligible to participate in the study. Outpatients and patients hospitalized at the facilities participating in this study were eligible.

Subjects must satisfy all of the following criteria for inclusion in the study.

1. The presence of gastrointestinal cancer, regardless of type, must be histologically confirmed.

2. Asymptomatic deep vein thrombosis (DVT) or PE, without restrictions on location, was newly developed during systemic chemotherapy for gastrointestinal cancer. Patients with symptomatic thrombosis* were ineligible. In addition, patients who develop thrombosis during systemic chemotherapy as postoperative adjuvant chemotherapy could be registered.

* DVT includes symptoms such as erythema, warmth, pain, swelling, tenderness, and pain during dorsiflexion of the legs, and PE includes symptoms such as sudden dyspnea, tachypnea, tachypnea, and fainting. Signs such as hypotension and hypoxemia were indicative of “symptomatic” thrombosis.

3. Pre-screening confirmed the absence of thrombosis before a diagnosis of new-onset asymptomatic VTE was rendered. In pre-screening, a serum d-dimer level of ≤1.2 µg/mL was considered “no thrombosis.” In that case, computed tomography (CT) and venous echography of both lower extremities were not required. Conversely, if the serum d-dimer level exceeded 1.2 µg/mL, either thoracoabdominal CT or venous echography of both lower extremities was needed to confirm the absence of thrombosis. Venography was not essential for the imaging diagnosis of thrombosis, including pre-screening.

4. Age of 20–90 years at the time of providing consent.

5. Provision of informed consent.

6. Meeting the following criteria for major organ function based on an examination performed within 2 weeks before registration:

(A) Neutrophil count ≥ 1.5 × 10^3^ /μL

(B) Platelet count ≥ 7.5 × 10^4^ /μL

(C) Hemoglobin ≥ 7.0 g/dL

(D) Total bilirubin ≤ 1.5 mg/dL

(E) Aspartate aminotransferase less than twice the institutional reference value

(F) Alanine aminotransferase less than twice the institutional reference value

(G) Serum creatinine < 1.5 mg/dL

7. Eastern Cooperative Oncology Group performance status of 0–2.

8. Expected survival of at least 3 months from the date of registration.

# **Exclusion criteria**

Subjects who met any of the following criteria were not eligible for enrollment.

1. Symptomatic deep vein thrombosis (DVT) with symptoms associated with thrombosis such as erythema, warmth, pain, swelling, tenderness, and pain during dorsiflexion of the foot.

2. Symptomatic PE with symptoms associated with thrombosis such as sudden dyspnea, tachypnea, tachycardia, syncope, hypotension, and hypoxemia.

3. History of thrombectomy, inferior vena cava filter insertion, or thrombolytic drug administration to treat DVT or PE that developed prior to enrollment.

4. The attending physician determined that treatment was required for thrombosis in the acute phase.

5. Thrombosis pre-screening (inclusion criterion 3) was not been performed before the onset of asymptomatic VTE.

6. Scheduled oral vitamin K antagonist (VKA) administration.

7. Administration of an anticoagulant (small-molecule heparin, unfractionated heparin, or fondaparinux) for more than 72 h before enrollment to treat DVT or PE or the receipt of more than one dose of a VKA.

8. Active bleeding (e.g., intracranial hemorrhage, retroperitoneal hemorrhage, intracystic hemorrhage, ulcerative hemorrhage), mild epistaxis, gingival bleeding, or gross hematuria at the time of the registration.

9. Presence of any of the following VTE risk factors within 3 months before enrollment:

― Four days or more of bed rest, pregnancy, childbirth, heart failure, myocardial infarction, acute bacterial endocarditis, spinal cord injury, lower-limb paralysis, fracture, lower-limb gypsum bandage fixation, lower-limb varicose veins, severe trauma, vasculitis, nephrosis syndrome, severe infection, inflammatory bowel disease, myeloproliferative disorders, varicose veins, oral contraceptive use, hormone therapy, hemostatic agent use, red blood cell hematopoietic factor preparation use

10. Protein C deficiency, protein S deficiency, antithrombin deficiency, hyperhomocysteinemia, or antiphospholipid antibody syndrome.

11. Uncontrolled hypertension (systolic blood pressure > 170 mmHg or diastolic blood pressure > 100 mmHg despite hypertensive treatment).

12. Expected continuous use of non-steroidal anti-inflammatory drugs other than aspirin for at least 4 days per week during the study period.

13. Expected continuous receipt of an aspirin dose exceeding 100 mg/day or two antiplatelet drugs (aspirin plus one antiplatelet drug or two other antiplatelet drugs) during the study period.

14. Systemic administration of the antiarrhythmic drug dronedarone at the time of registration.

15. Creatinine clearance (CrCl) level of less than 30 mL/min as calculated using the Cockcroft–Gault formula.

16. Presence of coelomic fluid (e.g., pleural effusion, ascites, pericardial fluid) requiring treatment.

17. Arterial thromboembolism, such as unstable angina and myocardial infarction, cerebral infarction, within 24 weeks before registration (excluding asymptomatic lacunar infarction).

18. History of severe drug hypersensitivity.

19. Severe liver disease (e.g., acute hepatitis, chronic active hepatitis, cirrhosis), alanine aminotransferase level more than twice the standard value, or total bilirubin leevl of more than 1.5 mg/dL, which is the upper limit of the normal value.

20. Local or systemic active infection requiring treatment or fever in a patient with suspected infection.

21. New York Heart Association Cardiac Function Classification grade II or higher heart failure or severe heart disease.

22. Estimated life expectancy of fewer than 3 months.

23. Deemed inappropriate as a research subject by the principal investigator.

# **Primary outcomes**

The primary outcome in this study was the incidence of major bleeding (MB) or clinically relevant non-major bleeding (CRNMB) during the first 3 months of edoxaban administration. In this study, the primary endpoint (safety) was evaluated in a population of patients who received the therapeutic research drug at least once. Because it was permissible to stop taking the research drug in this study if the research subject discontinues treatment during the observation period, the period from the last dose to 3 days after the previous dose was defined as the on-treatment period. Next, the incidence and 95% confidence interval were calculated for MB and CRNMB both separately and collectively. If the width of the confidence interval was within 9%, it was judged that the event rate could be estimated with sufficient accuracy.

# **Secondary outcomes**

1. Thrombus reduction rate 3 months after the start of edoxaban treatment.

2. Time to blood clot disappearance.

3. Total edoxaban dose.

4. Combined endpoint of new symptomatic deep vein thrombosis (DVT) development/symptomatic non-fatal pulmonary thromboembolism (PE), and death after the start of edoxaban treatment.

5. Event-free survival, defined as the proportion of subjects free of recurrent VTE and death.

6. Occurrence of hemorrhagic events/adverse events during the entire observation period.

7. Subgroup analysis (safety and efficacy) with and without dose adjustment criteria (edoxaban 60 and 30 mg).

8. Subgroup analysis by cancer type (safety and efficacy).

9. Subgroup analysis (safety and efficacy) according to renal function (creatinine clearance: ≤50 mL/min vs. >50–80 mL/min vs. >80 mL/min).

10. Subgroup analysis (safety and efficacy) by weight (≤60 kg vs. >60 kg).

11. Subgroup analysis (safety and efficacy) by age (<75 years vs. ≥75 years).

# **Index event and outcome event definitions**

### **Bleeding events: MB**

An MB event was confirmed as clinically overt bleeding meeting at least one of the following criteria:

a) Fatal bleeding

b) Bleeding in a critical area or organ such as:

- Retroperitoneal
- Intracranial
- Intraocular
- Intraspinal
- Intra-articular
- Pericardial
- Intramuscular with compartment syndrome

c) A clinically overt bleeding event

- - A decrease in hemoglobin levels of 2.0 g/dL over 24 h
  - Transfusion of ≥2 units of packed red blood cells

## **Categories of MB**

Category 1. Bleeding events presenting without any clinical emergency

Category 2. All bleeding events requiring certain measures without necessitating urgent care that could not be classified to any of the other three categories

Category 3. Bleeding events resulting in a significant medical emergency; e.g. hemodynamic instability, cerebral major bleeding presenting with neurologic symptoms

Category 4. Bleeding events that were fatal before or almost immediately after entering the hospital

### **Bleeding events: CRNMB**

A bleeding event was classified as a CRNMB event if it was an overt event (i.e., is symptomatic or visualized by examination) not meeting the criteria for MB that required medical attention, caused discomfort such as pain, or impaired activities of daily living.

- Overt bleeding requiring medical intervention
- Unscheduled contact with a physician
- Interruption of edoxaban
- Discomfort or impairment of activities of daily living

## **Nuisance (not clinically relevant) bleeding events**

Other overt bleeding events that did not fulfill the criteria of MB or CRNMB were classified as nuisance bleeding events.

## **Evaluation of efficacy**

Contrast-enhanced CT was used to compare the thrombus reduction rate between before and after 3 months of study drug treatment. The evaluation categories were as follows:

- ***Exacerbation***: New thrombus formation or apparent increase in the thrombus volume versus baseline. An increase in the thrombus diameter of 4 mm or more was defined as an increase.
- ***Improvement***: Apparent reduction of blood clots compared to baseline. Shrinkage was indicated by a ≥50% reduction in the thrombus volume.
- ***Disappeared***: A thrombus present at baseline can no longer be completely identified.
- ***No change***: Any state not qualifying for the other three categories.

As a central review for evaluating thrombus, a single independent radiological specialist, without additional clinical information, reviewed the imaging data that triggered the diagnosis of cancer-associated thrombosis and the subsequent imaging data.
