# Supplementary figures and images for: Feasibility of edoxaban for asymptomatic cancer-associated thrombosis in Japanese patients with gastrointestinal cancer: ExCAVE study
Source: BMC Cancer. 2022 Dec 16;22:1322. doi: 10.1186/s12885-022-10403-y (PMC9757916; doi:10.1186/s12885-022-10403-y)

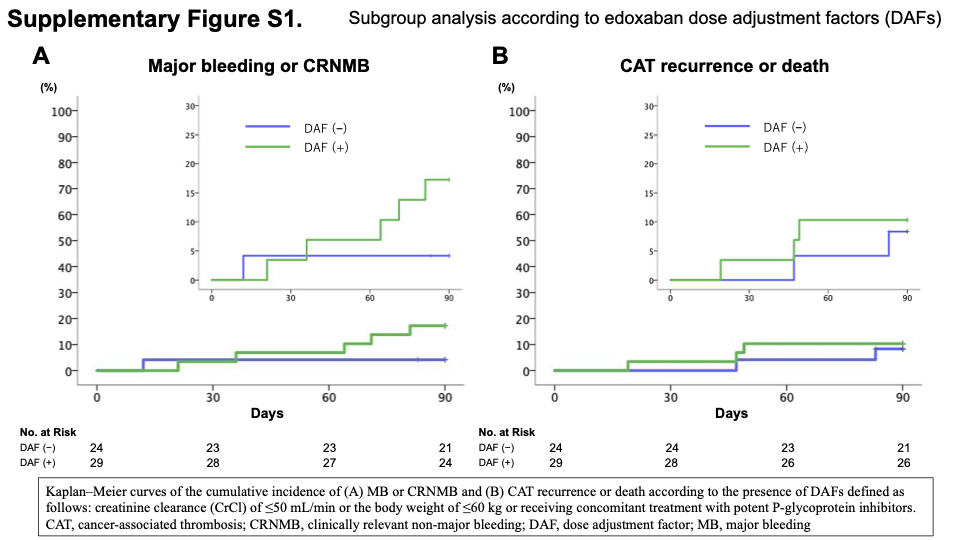

Supplement: Supplementary file 2 — Additional file 2. [file 12885_2022_10403_MOESM2_ESM.tiff]

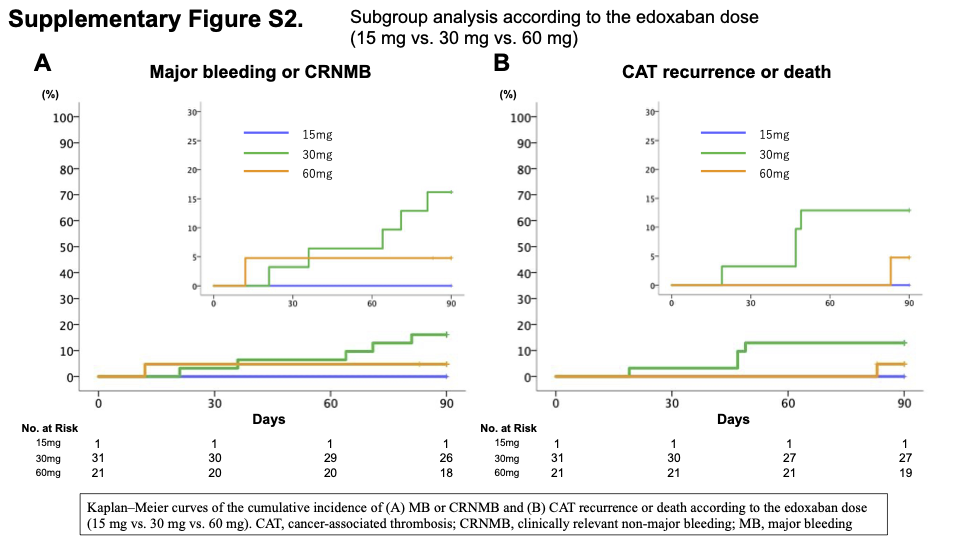

Supplement: Supplementary file 3 — Additional file 3. [file 12885_2022_10403_MOESM3_ESM.tiff]

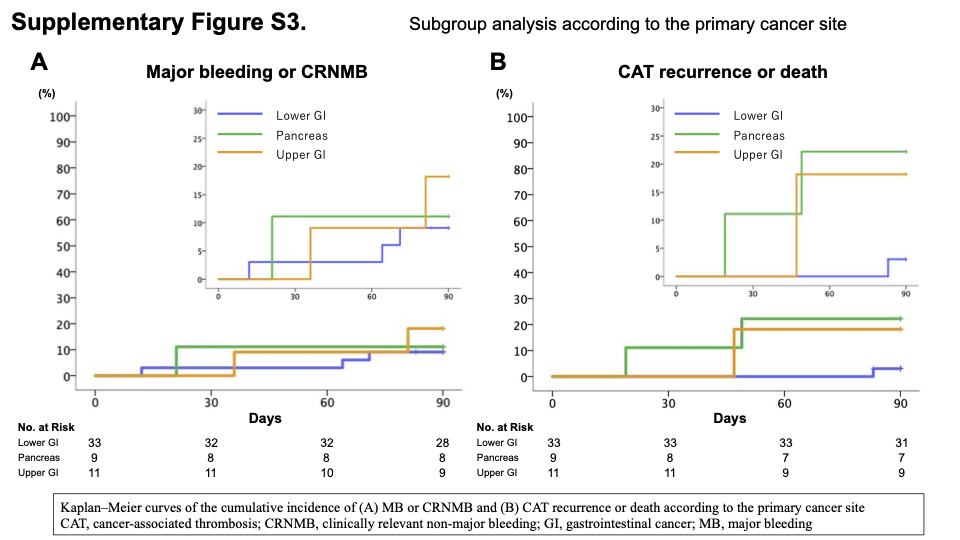

Supplement: Supplementary file 4 — Additional file 4. [file 12885_2022_10403_MOESM4_ESM.tiff]

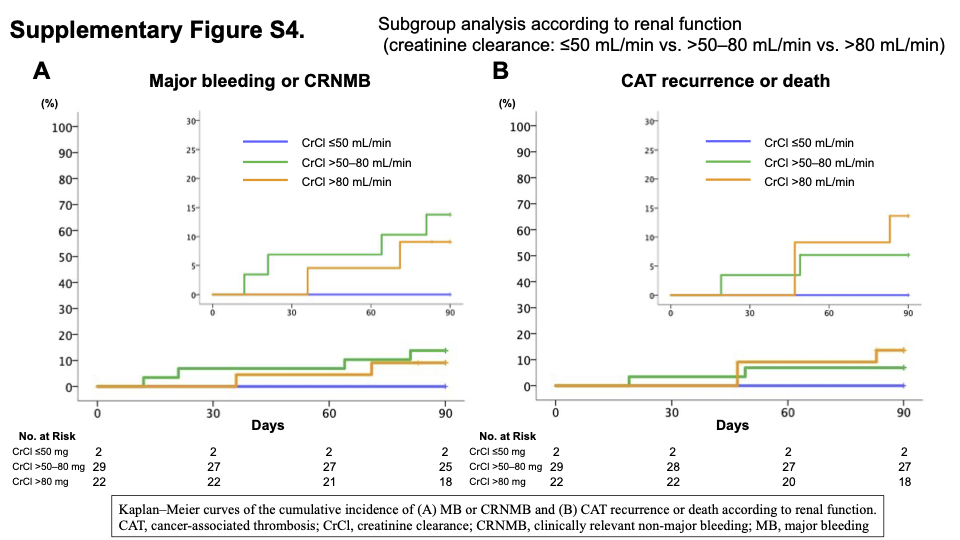

Supplement: Supplementary file 5 — Additional file 5. [file 12885_2022_10403_MOESM5_ESM.tiff]

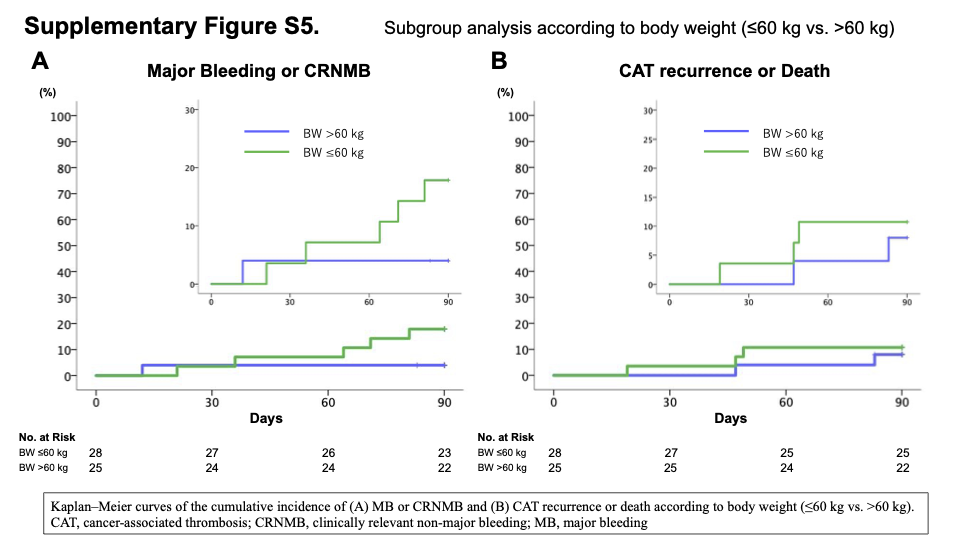

Supplement: Supplementary file 6 — Additional file 6. [file 12885_2022_10403_MOESM6_ESM.tiff]

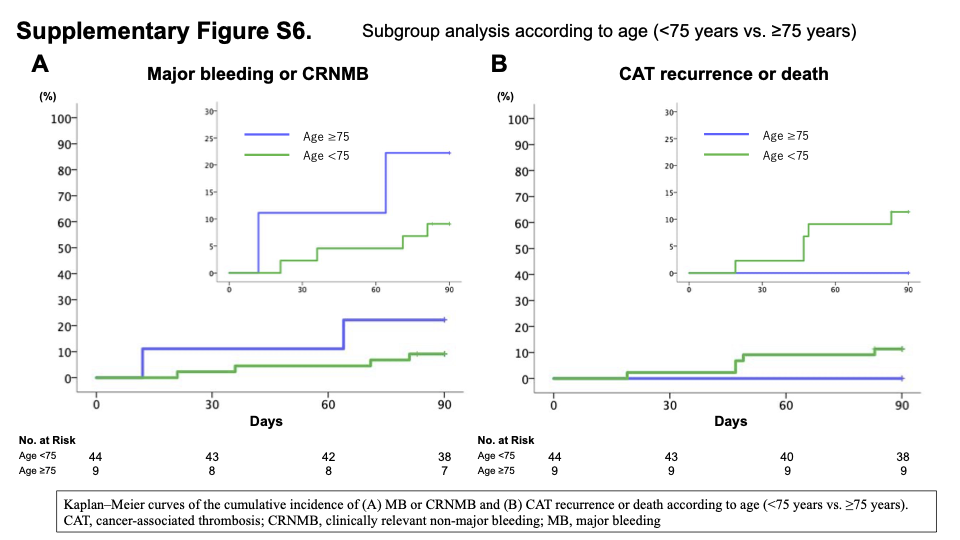

Supplement: Supplementary file 7 — Additional file 7. [file 12885_2022_10403_MOESM7_ESM.tiff]
